# Fever of unknown origin as a presentation of colonic inflammatory myofibroblastic tumor in a 36-year-old female: A case report

**DOI:** 10.3892/ol.2014.1942

**Published:** 2014-03-05

**Authors:** RU ZHOU, JIANBIN XIANG, ZONGYOU CHEN, ZHENYANG LI, JUN HONG

**Affiliations:** Department of General Surgery, Huashan Hospital, Fudan University, Shanghai 200040, P.R. China

**Keywords:** inflammatory myofibroblastic tumor, colonic tumor, fever of unknown origin, gastrointestinal stromal tumor

## Abstract

Inflammatory myofibroblastic tumor is a rare type of lesion that mimics malignancy and has various clinical manifestations. The current study presents a 36-year-old female with a colonic mass, which closely resembled a stromal tumor during imaging. The patient experienced intermittent fever and slight abdominal pain for one month. The fever remained at ≤38.5°C until the day of surgery. The patient underwent a right hemicolectomy and the preoperative fever disappeared and did not recur until the patient was discharged.

## Introduction

Inflammatory myofibroblastic tumor (IMT), also known as an inflammatory pseudotumor, is an uncommon mesenchymal neoplasm of intermediate malignant potential. IMT usually presents in children and young adults and has been found to occur in numerous locations, including the lungs, mesentery, omentum, soft tissues of the head and neck, retroperitoneum, liver and urinary bladder (1,2,3). However, IMT of the digestive tract is rare and only a few cases have previously been reported (2,3). The current report presents one case of a 36-year-old female with an IMT involving the colonic wall. The patient provided written informed consent.

## Case report

A 36-year-old female was hospitalized at the Department of Infection (Huashan Hospital, Shanghai, China) due to fever of unknown origin. The patient experienced an intermittent fever of ≤38.5°C, which was preceded by feeling cold, however, this was not accompanied by marked shivering or sweating; in addition, the patient experienced slight right abdominal pain for one month. Non-steroidal anti-inflammatory agents, such as indomethacin, alleviated the fever. However, the body temperature of the patient gradually increased again several hours later. The tuberculin skin test was negative for *Mycobacterium tuberculosis*. The body temperature remained abnormal following antibiotic medications, including cephalosporin and penicillin, which were administered at the First Affiliated Hospital of Nanchang University (Nanchang, China) where the patient was initially admitted. The patient had no history of serious illness, surgery or hospitalization.

On admission to the Huashan Hospital, no abnormalities were detected on physical examination and a laboratory evaluation revealed almost normal blood routine test results (hemoglobin levels, 11.5 g/dl; white blood cell count, 8.57×10^9^/litre; neutrophil count, 68%; and platelet count, 358×10^9^/litre). The erythrocyte sedimentation rate and C-reactive protein levels were elevated to 120 mm/h and 159 mg/l, respectively. Electrolytes, urinalyses and renal function tests were normal, however, liver function was abnormal (alanine transaminase, 216 U/l; aspartate transaminase, 152 U/l; γ-glutamyltransferase, 171 U/l; alkaline phosphatase, 281 U/l; and lactate dehydrogenase, 314 U/l), as a result of long-term use of antibiotics. In addition, the patient’s antistreptolysin O and anti-Jo-1 antibodies were identified as positive. Serum markers for hepatitis B and C, anti-dsDNA, antinuclear antibodies and antineutrophil cytoplasmic antibodies were all negative. No pathogens, including parasites, were observed in the stool samples.

**Figure 1:**
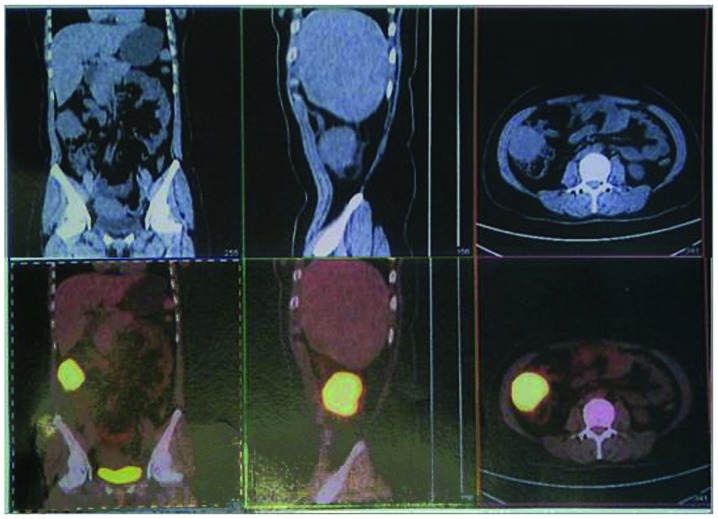
Positron emission tomography/computed tomography scan of the abdomen revealed a large solid mass with hypermetabolism of fluorodeoxyglucose. The mass is highlighted in yellow and was close to the liver.

During hospitalization, the patient underwent ultrasonography of the abdomen that showed a mass lesion with abnormal echo in the right lower quadrant of the abdomen, which was considered to be an inflammatory mass. This mass was confirmed by a computed tomography (CT) scan, which localized the tumor to the ascending colon and was considered to be a stromal tumor. In addition, a positron emission tomography/CT scan showed an exogenous tumor at the ascending colon (close to the hepatic flexure), which was associated with the increased metabolism of fluorodeoxyglucose (Fig. 1). The radiologist diagnosed the tumor as a stromal tumor. A colonoscopy was performed and no neoplasm was evident, however, there was a small quantity of colon polyps.

The fever remained between 38.5 and 39°C up to and including the day of surgery. The intraoperative examination revealed an irregularly margined, round and rubbery mass, which appeared to arise from the ascending colon (close to the hepatic flexure). The mass was tightly adhered to the side of the peritoneum, transverse colon and a section of the great omentum. A right hemicolectomy was performed to include the region of the tumor.

**Figure 2:**
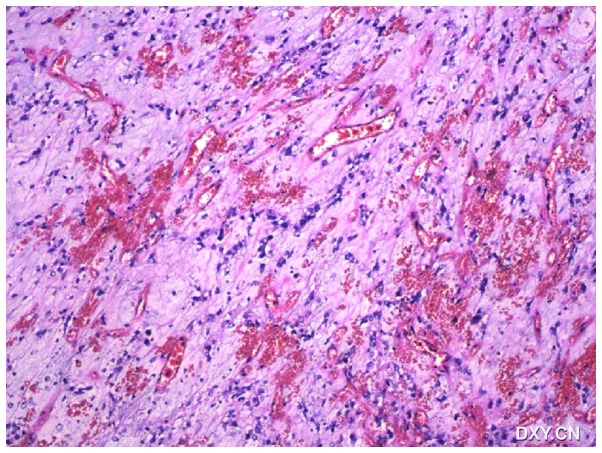
Tumor was principally composed of spindle or plump cells that exhibited round or elongated nuclei, prominent nucleoli with hypercellularity and mild nuclear atypism (stain, hematoxylin and eosin; magnification, ×100).

Gross examination of the primary tumor revealed an ill-defined, smooth, glistening mass that infiltrated the colonic wall (the intestinal mucosal surface was normal). The excised tumor measured 6.5×4.5×4.0 cm, and the cut surface of the primary tumor showed a white/gray lobulated mass and sections of the surface exhibited a cross-hatched appearance. Histologically, the tumor was characterized by fibrosis and proliferation of the spindle cells. In addition, polymorphous inflammatory infiltration of the plasma cells, histiocytes and lymphocytes with fibroblasts were noted (Fig. 2). The pattern of growth was infiltrative, with the whole thickness of the colonic wall infiltrated by the proliferating spindle cells. Immunohistochemistry of the atypical tumor cells revealed positive immunoreactivity for smooth muscle actin (SMA) and vimentin, and focal immunoreactivity for cluster of differentiation (CD)34, Ki-67, leukocyte common antigen (CD45), desmin and S-100; however, staining for cytokeratin, CD117 and discovered on GIST (DOG)-1 protein was negative.

Furthermore, the gene test showed no mutations in exons 9, 11, 13 or 17 of the c-KIT gene, or exons 12 or 18 of the platelet-derived growth factor receptor A gene. The results of the gene test combined with the negative immune reactivity of DOG-1 and CD117 excluded the diagnosis of a gastrointestinal stromal tumor. Leiomyoma and schwannoma were also excluded, due to the unclear positive immunoreactivity for desmin and S-100.

The preoperative fever subsided and did not recur until the patient was discharged. The patient was considered to be healthy without evidence of recurrence during the one year following surgery.

## Discussion

IMT is a clinical and pathological disease entity (1). It is an inflammatory lesion with unknown etiology that originates from any site of the body (2). The term IMT is used to denote a histologically similar group of tumors, which are characterized by spindle cell proliferation with a fibroinflammatory appearance. IMTs have previously been reported under a variety of additional descriptive terms, such as inflammatory fibroid polyp, inflammatory pseudotumor, plasma cell granuloma and pseudosarcomatous myofibroblastic proliferation (2,3). Despite an apparently benign morphological nature, certain cases of IMT have been reported to have a malignant clinical course, including a locally aggressive growth and a tendency to recur following complete resection (4). Therefore, it is difficult to confirm an accurate diagnosis prior to surgery (5).

Previously, Coffin *et al* (2) described the following three histological patterns of IMT: i) Myxoid vascular pattern resembling nodular fasciitis; ii) compact spindle cell pattern with a fascicular or storiform cellular arrangement; and iii) hypocellular collagenized pattern resembling a scar or desmoid fibromatosis. In any case of IMT, a combination of all of these patterns may be present or any one pattern may be predominant. Immunohistologically, the spindle cells have been identified to be reactive against antibodies to vimentin, SMA and muscle-specific protein in the majority of cases (2), which is comparable to the observations of the present study.

The majority of extrapulmonary IMTs, including colonic IMTs, are successfully curable by surgical resection without the development of recurrence (3,6). The prognosis of an IMT of the digestive tract is usually good (3), however, certain cases of IMT that originate from other organs may show recurrence or even metastasis following surgery (7–11). The tendency for local recurrence appears to be associated with the initial site of the IMT (3). For this reason, numerous authors have recommended complete surgical resection of the lesion as the first treatment of choice for IMTs (3,9,12,13).

In conclusion, IMT is an extremely rare lesion that mimics malignancy and is accompanied by various clinical manifestations; however, they are often benign lesions and are surgically curable. Therefore, an IMT must be considered as the possible cause for an abdominal mass that is accompanied by various clinical manifestations. Thus, all patients must undergo a careful long-term follow-up due to the unknown risk of recurrence.
